# The interaction between miRNAs and hazardous materials

**DOI:** 10.1016/j.ncrna.2023.06.005

**Published:** 2023-06-28

**Authors:** Soudeh Ghafouri-Fard, Hamed Shoorei, Shahram Dabiri Oskuei, Bashdar Mahmud Hussen, Snur Rasool Abdullah, Mohammad Taheri, Elena Jamali

**Affiliations:** a Department of Medical Genetics, School of Medicine, Shahid Beheshti University of Medical Sciences, Tehran, Iran; b Department of Anatomical Sciences, Faculty of Medicine, Birjand University of Medical Sciences, Birjand, Iran; c Clinical Research Development Unit of Tabriz Valiasr Hospital, Tabriz University of Medical Sciences, Tabriz, Iran; d Department of Clinical Analysis, College of Pharmacy, Hawler Medical University, Kurdistan Region, Iraq; e Medical Laboratory Science, Lebanese French University, Kurdistan Region, Erbil, Iraq; f Institute of Human Genetics, Jena University Hospital, Jena, Germany; g Urology and Nephrology Research Centre, Shahid Beheshti University of Medical Sciences, Tehran, Iran; h Loghman Hakim Hospital, Shahid Beheshti University of Medical Sciences, Tehran, Iran

**Keywords:** miRNA, Hazardous material, Cancer

## Abstract

Toxic agents are broadly present in the environment, households, and workplaces. Contamination of food and drinking water with these agents results in entry of these materials to the body. The crosstalk between these agents and microRNAs (miRNAs) affects pathoetiology of several disorders. These agents can influence the redox status, release of inflammatory cytokines and mitochondrial function. Altered expression of miRNA is involved in the dysregulation of several pathophysiological conditions and signaling pathways. These molecules are also implicated in the adaption to environmental stimuli. Thus, the interactions between miRNAs and toxic materials might participate in the hazardous effects of these materials in the body. This review describes the effects of the toxic materials on miRNAs and the consequences of these interactions on the human health.

## 1 Introduction

Hazardous materials are radioactive substances and/or chemical compounds which can be harmful to individuals or animals and the environment upon exposure [1]. As chemical materials, they fall into a category such as cadmium, lead, mercury, arsenic, chromium, and asbestos [[2], [3], [4], [5]]. The United States Environmental Protection Agency (USEPA) has considered some of them to be probable human carcinogen agents. These materials are found in drinking water [6], air [7], and food [8], and therefore they could be absorbed via dermal contact, ingestion, and/or inhalation, leading to damage to body organs such as the lungs, kidneys, and liver. Different levels of poisoning occur after exposure to such substances. For example, arsenic is a well-known carcinogenic agent and is strongly linked to the development of lung, bladder, liver, and kidney cancers [9,10]. Or, asbestos fibers, including chrysotile, are highly associated with the development of lung cancer, mesothelioma, and pulmonary fibrosis [11,12].

A number of studies have shown that development of several diseases such as hypertension, gastrointestinal disorders, and osteoporosis is resulted from long-term exposure to these materials [[13], [14], [15], [16], [17]]. It has been also reported that some of these hazardous agents such as mercury, lead, and cadmium could pass from the placenta and cause a disruption in the normal process of fetal development [18,19].

In recent years, scientists have focused on a wide range of molecular alterations and mechanisms involved in hazardous material-related diseases [20,21]. Their results have shown that these mentioned materials could affect normal cell function and lead to cell death via a number of mechanisms including DNA methylation, inflammation, oxidative stress, autophagy, and apoptosis [22,23]. However, among these mechanisms, it has been reported that microRNAs (miRNAs) are associated with multiple organ injuries [24,25]. miRNAs are categorized as a form of the molecules of non-coding RNAs with nearly ∼22 nucleotides in length [26,27]. Although they are not involved in protein coding, they could modify target mRNAs via the posttranscriptional mechanism. Both genetic and epigenetic mechanisms could also regulate miRNAs expression [28]. In this regard, for example, in human bronchial epithelial cells (HBECs) exposed to arsenic, elevated promoter methylation has led to suppression of miR-200. Moreover, arsenic has caused malignant transformation via altering epithelial-mesenchymal transition (EMT) signaling pathways [29]. Moreover, in lung cancer asbestos could alter the miRNA expression, where the expression of some miRNAs such as miR-202, miR-605, and miR-939 decreased, while the expression of several miRNAs, such as miR-96, let-7d/e, and/or miR-374a, increased [30]. In this review, we investigated the interaction of miRNAs and some important hazardous materials.

## 2 Interaction between miRNAs and arsenic compounds

Arsenic compounds have been used for treatment of leukemia. For instance, arsenic trioxide (As [2]O(3), ATO) has been used for treatment of acute promyelocytic leukemia. Gao et al. have assessed possible synergy between miR-15a/16-1 and ATO in K562 cells. They have reported that combination of miR-15a/16-1 and ATO induces growth suppression and apoptosis in these Bcr-Abl positive leukemic cells. Mechanistically, apoptosis is induced through regulation of mitochondrial functions. In fact, this process involves release of cytochrome *c* and loss of mitochondrial transmembrane potential. Yet, ATO and/or miR-15a/16-1 could not affect expression of Bcr-Abl in these cells. Besides, miR-15a/16-1 and ATO could induce apoptosis in Bcr-Abl negative leukemic cell lines and primary leukemic cells in a synergic manner [31]. Another study has shown that anti-miR-21 oligonucleotide (AMO-miR-21) and ATO inhibit growth of K562 cells and induce apoptosis and G1 arrest in these cells. Mechanistically, AMO-miR-21 induces sensitivity to ATO through induction of apoptosis via up-regulation of PDCD4 levels [32].

**Fig. 1:**
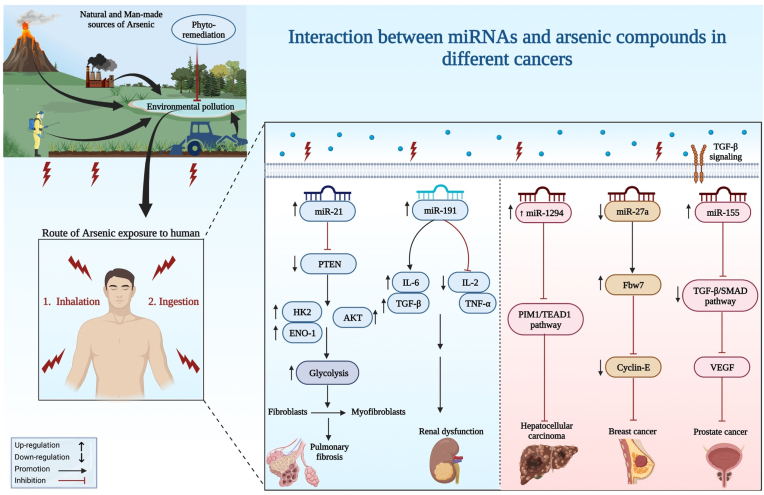
A schematic representation shows the routes of arsenic exposure in humans and miRNA interaction signaling with arsenic in different types of cancer.

miRNAs expression has also been found to be altered after arsenic exposure participating in the arsenic-induced multiorgan damage. Upregulation of miR-155 has been shown to be involved in the arsenic induced skin injury. Moreover, expression levels of miR-21 and miR-145 have been found to be associated with liver damage, while levels of miR-191 have been linked with kidney damage (Fig. 1). Furthermore, miR-155 has can be used as a diagnostic marker for arsenic-induced skin damage. Moreover, miR-21 and miR-145 have been suggested as diagnostic markers for liver damage. Finally, miR-191 can be used as a diagnostic marker for kidney damage [33]. Table 1 summarizes the results of studies that evaluated interactions between miRNAs and arsenic compounds.

**Table 1:** Interaction between miRNAs and arsenic compounds.

miRNA	Human/animal Study	Dose	Cell line	Dose	Targets	Observations	Ref
miR-15a, miR-16-1 (−)			Raji, HL60, RPMI8226, K562, U937	1 μM, for 48 h	Caspase-3, Cytochrome-c	In K562 cells, both mentioned miRNAs could sensitize apoptosis induced by ATO.	[31]
miR-21 (−)	–	–	K562	0.5–4.0 μM, 4 h	PDCD4	The sensitization of K562 cells exposed to ATO increased because of the anti-miR-21 oligonucleotide.	[32]
miR-21, miR-200b, miR-191, miR-155, miR-145 (Up)	Human	–	–	–	–	Exposure to arsenic by elevating the expression of mentioned miRNAs could lead to multiorgan (such as skin, liver, and kidney) damage.	[33]
miR-21, miR-155, miR-200b (Up)	miR-21 knockout mice, wild-type mice	20 ppm, 6 months	HBE, MRC-5	0–8 μM, 24 h	PTEN, AKT, α-SMA	In the pulmonary fibrosis model induced by NaAsO₂, miR-21 via glycolysis could elevate the differentiation of myofibroblast.	[34]
miRNA profile, miR-29a (−)	–	–	HepG-2	0–6 μM, 24 h	Wip-1, PPM1D	In HepG-2 cells, miR-29a could mediate ATO induction of cell death.	[35]
miR-27a (Down)	–	–	MDA-MB-231, SK-BR-3	0–14 μM, for 24 h	Fbw7, Cyclin-E	In breast cancer cells, ATO via suppressing miR-27a could inhibit tumorigenesis.	[36]
miR-31 (Down)	–	–	BEAS-2B	0–10 μM, 48 h, 2 μM, 6 weeks	SATB2	Arsenic via reducing miR-31 and elevating SATB2 could induce malignant transformation in BEAS-2B cells.	[37]
miR-34a, miR-133b, (Down)	Human	–	U251, SH-SY5Y	5 or 10 μM, 24 h	hERG	miR-133b via sponging the hERG could lead to apoptosis.	[38]
miR-98 (Up)	SD rats	0.4 mg/kg, 2 weeks	A549	0–10 μM, for 48 h	Stat3, α-SMA, E-cadherin, Bax, Bcl-2	Pulmonary fibrosis induced by bleomycin could be decreased after ATO treatment via increasing miR-98 expression.	[39]
miR-126 (Down), miR-155 (−)	Human (Mexican children)	–	–	–	–	Inorganic arsenic could change the expression of miR-126.	[40]
miR-148a (Up)	–	–	Multiple drug-resistant (MDR) Bel-7402	0–3.5 μM, 24 h	NF-κB	ATO via demethylating miR-148a and suppressing the NF-κB could promote cell sensitivity to chemotherapeutic agents.	[41]
miR-155 (Up)	Mice	2 mg/kg, 2/week, 2 months	PC-3, HUVEC, LNCaP	0–4 μM, 48 h	VEGF, TGF-β, SMAD	In prostate cancer, ATO could induce anti-angiogenic effects via elevating miR-155 and suppressing the TGF-β/SMAD pathway.	[42]
miR-155 (Up)	–	–	A549, A549R	0–30 μM, 0–150 μM, 72 h	HO-1, Nrf-2, NQO1, Bax, NQO1	miR-155 via suppressing apoptosis and enhancing Nrf2 could mediate cell resistance to ATO.	[43]
miR-184, miR-576-3p, (−)	Human	59–172 ppb	–	–	–	In skin lesions (west Bengal people) induced by arsenic, the expression of miRNAs could change.	[44]
miR-190 (Up)	–	–	BEAS-2B, A549	0–20 μM (6 h),	PHLPP, AKT, Talin-2	Exposed cells to AsCl3 by activating AKT, elevating miR-190, and suppressing PHLPP could lead to carcinogenesis.	[45]
miR-191 (Up)	Human	–	–	–	IL-2/6, TGF-β, TNF-α	miR-191 via activating inflammatory response could lead to renal dysfunction induced by coal-burning arsenic.	[46]
miR-199a-5p (Down)	Female CrTac: NCrFoxn1^nu^ mice	–	AsT, BEAS-2B	0–2 μM (24 h),	HIF-1α, COX-2	Overexpression of miR-199a via targeting COX-2 and HIF-1α could suppress angiogenesis in bronchial epithelial cells.	[47]
miR-203 (−)	–	–	K562	1.25–20 μg/mL, 48 h	Caspase-3/9, Cytochrome-c	hsa-miR-203 could increase leukemia cell sensitivity to ATO.	[48]
miR-222 (Up)	–	–	BEAS-2B	1 μM, 26 weeks	ARID1A, PTEN, AKT	Administration of anti-miR-222 could inhibit tumor growth induced by arsenic.	[49]
miR-301a (Up)	Athymic nude mice	–	BEAS-2B, BEAS-2B–As	0–10 μM 12 h	SMAD4, IL-6, STAT3	NaAsO₂ via increasing miR-301a could lead to malignant transformation of BEAS-2B cells.	[50]
miR-425-5p (Down)	C57BL/6J mice	(0, 1, or 10 ppm), 3 months	HUVECs, 293T	1–40 μM, for 24–48 h	CCM3, Notch, VEGF/p38	NaAsO_2_ had anti-angiogenesis effects in HUVECs.	[51]
miR-539 (Down)	Human, Male athymic nu/nu mice	5 mg/kg, one injection/3 days, 18 days	Primary human hepatocytes, HepG2, Hep3B, Huh7, PLC/PRF/5, Sk-Hep-1, PLC-ATR, HepG2-ATR	0–64 μM, for 48 h	Stat3, Bcl-2, Bcl-xL	In hepatocellular carcinoma, miR-539 could decrease cell chemoresistance induced by ATO.	[52]
miR-1294 (Up)	female BALB/C athymic nude mice		Huh6/7, HepG2, SMMC7721, Hep3B	0–12 μM, 48 h	TEAD1, PIM1, caspase-3, Bax, Bcl-2	In hepatocellular carcinoma, arsenic trioxide via upregulating miR-1294 and sponging PIM1/TEAD1 axis could inhibit tumor growth.	[53]
miR-2909 (Up)	–	–	PBMCs	0.5–2 μM, 72 h	Cyclin-D1, SP1 KLF4, NF-kB, BCL3	Arsenic via miR-2909 could mediate the regulation of Cyclin-D1.	[54]
miR-4665-3p (Down)	Human (gastric cancer patients)	–	MGC803, AGS, HCT116	133.36 (stock solution) μM, 24 h	GSE-1, VEGF, E-cadherin, Vimentin	As4S4 via increasing miR-4665-3p could suppress migration or invasion in gastric cancer cells.	[55]

### 2.1 Interaction between miRNAs and cadmium compounds

**Fig. 2:**
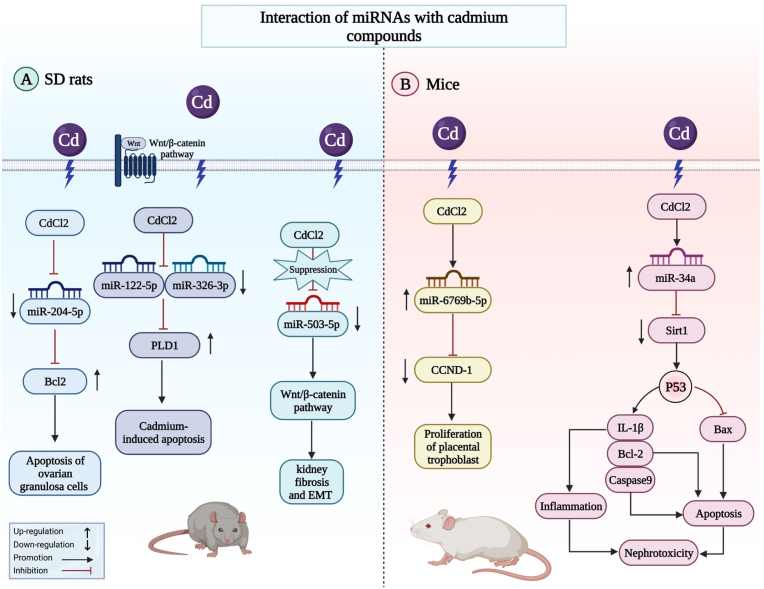
The interaction of miRNAs and cadmium compounds with signaling pathways in Sprague Dawley (SD) rats and mice animal models is shown schematically in the diagram.

Cadmium (Cd) is an important hazardous agent that has toxic effects on fish and aquatic animals. Expression profiling of miRNAs in cultured common carp (Cyprinus carpio L.) has shown differential expression of a number of miRNAs during Cd exposure. In fact, 7 and 16 miRNAs have been found to be up-regulated and down-regulated, respectively. miR-122, novel-miR6, miR-193a-3p and miR-27a-5p have been among differentially expressed miRNAs (Fig. 2a). Moreover, expressions of BAX, BAD, BAK, CASPASE9 and PIDD have been enhanced, while BCL2 expression has been reduced following Cd exposure. Changes in the expression levels of mentioned miRNAs might be involved in the oxidative stress-induced apoptosis following exposure to Cd [56]. Another study has demonstrated inflammation-related injury in the spleens of common carp following Cd exposure. In fact, 17 miRNAs have been up-regulated, while 6 miRNAs have been down-regulated. Theses miRNAs have been functionally related with NF-κB, Jak-STAT, MAPK, Th1 and Th2 cell differentiation, and Toll-like receptor signaling pathways [57]. Another experiments in rat ovarian granulosa cells has shown that Cd is cytotoxic to these cells affecting expression of a number of miRNAs. In fact, Cd-induced damage to these cells is mediated by mitochondrial apoptosis [57]. In mice animal model, miR-6769b-5p via sponging CCND-1 might involve in the proliferation of placental trophoblast treated with CdCl2. Furthermore, via modulating the miR-34a/Sirt1/p53 signaling pathway, cd can damage the kidneys of mice and can control the apoptosis and inflammation (Fig. 2b).

Table 2 shows the interaction between miRNAs and Cd.

**Table 2:** Interaction between miRNAs and cadmium compounds.

miRNA	Human/animal Study	Dose	Cell line	Dose	Targets	Observations	Ref
novel-miR-6, (Down), miR-27a-5p (Up), miR-122 (Down)	Common carp	0.275 mg/L, 1 month	–	–	Caspase-9, Bax, Bak, Bcl-2	In carp exposed to CdCl2, miRNA changes could be a biomarker.	[56]
17 upregulated miRNAs (such as miR-7-1-5p) and 6 downregulated miRNAs (such as miR-9-6-5p)	Juvenile fish	0.26 mg/L, 96h	–	–	MAPK, Jak/STAT, NF-κB, IL-4/13A, COX-2, PTGES,	CdCl2 in common carp spleens via targeting miRNA-mRNA networks could lead to inflammation.	[57]
miR-9a-5p, miR-29a-3p, miR-204-5p (Up)	Female SD rats	–	Rat ovarian granulosa cells, PC-12	0–20 μM, h	Bcl-2, Bax, Fas	Exposure to CdCl2 in rat ovarian granulosa cells via miR-204-5p and Bcl-2 could regulate apoptosis.	[57]
miR-21, miR-29b, (Up)	Human, SD rats	2.0 mg/kg, 2 weeks	–	–	–	The expression of miR-21 could be a potential biomarker for the dysfunction of the kidney.	[58]
44 upregulated (such as miR-21-5p, miR-3084c-3p), 54 downregulated (such as miR-455-3p, miR-193b-3p)	Male SD rats	0.6 mg/kg, 5 days a week,3 months	–	–	–	CdCl2 could induce nephrotoxicity and change the expression of miRNAs.	[59]
miR-25-3p (Down)	juvenile common carp	0.26 mg/L, 45 days	–	–	Hsp70/90, AMPK, PTEN, ULK1, mTOR, Atg-5/12, Beclin-1, LC3-II	Pollutants with CdCl2 in common carp could lead to autophagy and oxidative stress via miR-25-3p.	[60]
miR-26a, miR-155, (−)	Human	–	JEG-3	1–25 μM, 24–48 h	TGF-β, Smad-2/3	miRNAs could regulate the TGF-β pathway in trophoblast cells exposed to cadmium.	[61]
miR-26a (−)	–	–	JEG-3	0–25 μM, 48 h	TGF-β	The migration of placental trophoblast cells could be inhibited by exposure to CdCl2 via the miR-26a/TGF-β axis.	[62]
miR-27b-3p (Up), miR-877-5p (Down)	–	–	16HBE	5 ***μ***mol/L, 14 weeks	CCM2	Mentioned miRNAs could act as malignant transformation.	[24]
miR-30 family (a, b, d, c, e) (Down)	–	–	BEAS-2B, BEP2D	0–10 μM, 72 h	SNAIL, ZEB1, Vimentin, E-cadherin	In human lung epithelial cells, exposure to CdCl2 via suppressing miR-30 could promote SNAIL and fibrosis.	[63]
miR-33-5p (Down)	Hy-Line Brown laying hens	150 mg/kg, 3 months	–	–	BNIP3, LC3-I/II, Beclin-1, AMPK, AKT/mTOR, NF-kB/JNK,	In the chicken spleen, CdCl2 via regulating AMPK and miR-33 could induce autophagy, dependent on BNIP3.	[64]
miR-34a (Up)	Male Kunming mice	1.5 mg/kg, 1 month	–	–	Sirt1, p53, Bax, Bcl-2, IL-1β Caspase-9,	miR-34a via targeting p53 or Sirt1 could induce nephrotoxicity.	[65]
miR-34a (Up)	Male Wistar rats	10 mg/L	–	–	SIRT1, p53, IL-6, TNF-α, SREBP1/2,	The non-alcoholic fatty liver disease could be induced in an animal model by CdCl2.	[66]
miR-92a-2-5p (Down), miR-181b-5, (Up)	SD rats	0.5–8 mg/kg	granulosa cells	0–20 μM, 12 h	Bcl-2, Bax	miRNA profile could be changed in ovarian granulosa cells of rats exposed to CdCl2 during the prenatal period.	[67]
miR-92a-2-5p (Up)	Female SD rats	8 mg/kg, postnatal day [56]	granulosa cells, COV434	0–20 μM, 24 h	c-Myc, Bcl-2, DNMT3B, DNMT1, DNMT3A	After cadmium exposure, in rat ovarian granulosa cells, c-Myc could promote the transcription of miR-92a-2-5p.	[68]
miR-101 (Down)	–	–	HUVECs	0–80 μM, 0–36 h	COX-2, VEGF, eIF2α	miR-101 by sponging COX-2 could suppress angiogenesis induced by CdCl2 in HUVECs.	[69]
miR-122 (Down)	farmed tilapia	12 mg/L, 24 h	–	–	MT 3′UTR	miR-122 via sponging metallothionein gene could act against hepatic oxidants induced by CdCl2.	[70]
miR-122-5p, miR-326-3p, (Up)	Human, Male SD rats	0.6 mg/kg, 3 months	HK-2, NRK-52E	9.18 and 10 μM, 48 h	–	Both mentioned miRNAs could be an early detective biomarker for CdCl2 exposure.	[71]
miR-122-5p, miR-326-3p, (Up)	SD rats	0.6 mg/kg, 1.5 months	NRK-52E	10 μM, 48 h	PLD1	Both mentioned microRNAs via decreasing PLD1 could increase apoptosis in NRK-52E cells treated with CdCl2.	[72]
miR-143-3p (Up)	Human	–	hBMSCs	0–30 μM, 7–24 h	Wnt/β-catenin, ARL6, ALP, RUNX2, LEF1, TCF1	In hBMSCs exposed to CdCl2, miR-143-3p via targeting ARL6 could inhibit osteogenic differentiation.	[73]
miR-155, miR-181a, (Up)	Common carp	0.005–0.5 mg/L, 1 month	–	–	HO-1, NF-κB, TLR-4, IL-1β, IL-8/10	Both mentioned miRNAs via targeting HO-1 could lead to immunotoxicity in the carp's kidneys.	[74]
miR-155 (−), miR-221 (Down)	Human	–	–	–	IL-17, TNF-α	In workers exposed to CdCl2, there is an association between miRNAs and immune markers.	[75]
miR-217 (−)	Common carp	0.005–0.5 mg/L, 1 month	–	–	SIRT1, TLR-4, NF-kB, TRAF6	In common carp exposed to CdCl2, the miR-217/SIRT1 axis could lead to immunotoxicity.	[76]
miR-363-3p (Up)	Human (occupational chronic Cd poisoning)	–	HK-2, NRK-52E	0–64 μM, 48 h	PI3K, PARP, Caspase-3	miR-363-3p via suppressing PI3K could enhance cell death in the kidney.	[77]
miR-381 (Down)	–	–	HBEC	1 ***μ***M	EZH2, H3K27me3	In epithelial cells exposed to CdCl2, the miR-381/EZH2 axis could regulate the expression of the chloride channel.	[78]
miR-503-5p (Down)	SD rats	0.6 mg/kg, 6 or 12 weeks	NRK-52E	6–10 μM, 24 h	Wnt/β-catenin, α-SMA, Vimentin, Collagen1	CdCl2 could induce kidney fibrosis and EMT via suppressing miR-503-5p and promoting the Wnt/β-catenin pathway	[79]
miR-6769b-5p (Up)	Human, male and female CD-1 mice	2.5 mg/kg on the 15th gestational day	HTR-8/SVneo,	0–40 μM, for 24 h	CCND1, PCNA	miR-6769b-5p via sponging CCND-1 could be involved in the proliferation of placental trophoblasts treated with CdCl2.	[80]

### 2.2 Interaction between miRNAs and lead compounds

miR-106b-5p has been shown to be up-regulated by lead (Pb^2+^)-induced stress. miR-106b-5p has been shown to bind with the 3′-UTR of XIAP to down-regulate expression of XIAP. Inhibition of miR-106b-5p has been shown to reverse the decrease in IAP levels and cell viability in Pb^2+^-treated HT-22 and PC12 cells. Cumulatively, regulation of XIAP by miR-106b-5p might be associated with Pb neurotoxicity [81]. Another study has detected high levels of miR-155 and low levels of miR-126 in Pb exposed women. Moreover, authors have reported a significant simple positive relationship between blood lead levels and serum levels of miR-155. On the other hand, blood lead levels have been inversely correlated with serum miR-126 levels. Taken together, epigenetic changes might be linked with Pb exposure and its effects on health [82]. Besides, the interaction between miR-137 and EZH2 has been shown to contribute to the genome-wide redistribution of H3K27me3 which is responsible for Pb-associated memory impairment [83]. Table 3. Interaction between miRNAs and Pb in different contexts.

**Table 3:** Interaction between miRNAs and lead compounds.

miRNA	Human/animal Study	Dose	Cell line	Dose	Targets	Observations	Ref
miR-106b-5p (Up)	–	–	HT-22, PC12	0–100 μM, 48 h	XIAP	miR-106b-5p via targeting XIAP could inhibit cell viability.	[81]
miR-126 (Down), miR-155 (Up)	Human (Mexican women)	–	–	–	–	The expression mentioned miRNAs changed in Mexican women exposed to Pb.	[82]
miR-137 (Up)	Female SD rats	250 p.p.m.	PC12, primary hippocampal neurons	5 μM, 24 h	H3K27me3, EZH2, Wnt9b	Pb could lead to memory impairment via the miR-137/EZH2 axis.	[83]
miR-143-5p (Down)	C57BL/6 mice	10 mg/kg, 1 month	Renal interstitial fibroblasts	0–2 μM, 24 h	CCL20, Smad2/3, AKT, TGF-β1	Administration of miR-143-5p via sponging CCL20 could decrease renal fibrosis induced by Pb.	[84]
miR-146a (−)	C57BL/6 mice	200 mg/L, 15 days	BMECs	–	IRAK1, IL-2/8, PTX3	In the mammary gland, Pb could increase inflammation levels.	[85]
miR-148a (−)	Human	–	–	–		Exposure to Pb could make an association between the methylation of DNA and miR-148a.	[86]
miR-155 (Up)	Asian Carp	1–2 mg/L	–	–	ERK, p38, IL-6, TNF-α, IL-1β	In the carp's head kidney, miR-155 could induce inflammation in the MAPK-dependent pathway.	[87]
miR-155, miR-221, (Up)	Human (North-Western India)	–	–	–	–	Both mentioned miRNAs increased in workers exposed to Pb.	[88]
miR-378a-3p (Up)	male C57 mice	250–1000 mg/L, 12 weeks	HT22	10–40 μM, for 24 h	SLC7A11, GPX4	In a model of nerve injury induced by Pb, miR-378a-3p via sponging SLC7A11 could be involved in the induction of ferroptosis.	[89]

### 2.3 Interaction between miRNAs and asbestos

Comparison of miRNA signature between malignant pleural mesothelioma and benign asbestos-associated pleural effusion has led to identification of several up-regulated miRNAs in the former condition, among them being hsa-miR-484, hsa-miR-320, hsa-let-7a, and hsa-miR-125a-5p. These miRNAs have the potential to discriminate these two conditions [90]. Another study has reported down-regulation of miR-30d is in the pleural malignant mesothelioma cell line NCI–H2452, in the plasma samples of asbestos-exposed persons, and in mesothelial cells exposed to asbestos. Up-regulation of miR-30d could inhibit proliferation, migration, and invasion pleural malignant mesothelioma cells and enhance their apoptosis without affecting cell cycle. Moreover, it could decrease vimentin and TWIST1 levels, and increase CDH1 levels in NCI–H2452 cells. Thus, miR-30d is related to asbestos exposure and suppresses migration and invasion of NCI–H2452 cells through regulation of epithelial-mesenchymal transition [91]. Moreover, extracellular vesicle-levels of miR-103a-3p and miR-30e-3p have been shown to discriminate malignant pleural mesothelioma from past asbestos exposure [92]. Table 4 shows interactions between miRNAs and asbestos.

**Table 4:** Interaction between miRNAs and asbestos.

miRNA	Human/animal Study	Dose	Cell line	Dose	Targets	Observations	Ref
hsa-let-7a, miR-125a-5p, miR-320, miR-484, (Up)	Human (Malignant pleural mesothelioma patients)	–	–	–	WNT3, FGF9, TGFB2	The expression of miRNAs could be changed in pleural effusion induced by asbestos.	[90]
miR-30d (Down)	Human (pleural malignant mesothelioma patients)	–	NCI–H2452		Vimentin, CDH1, TWIST1	Exposure of NCI–H2452 cells to asbestos could suppress invasion or migration via miR-30d.	[91]
miR-30e-3p, miR-103a-3p, (Down)	Human (malignant pleural mesothelioma patients)	–	–	–	–	Extracellular vesicle miRNAs could be considered a biomarker of mentioned disease.	[92]
hsa-miR-98 (Down)	Human (Malignant pleural mesothelioma patients), GSE92636 database	–	–	–	–	Higher expression of miR-98 was associated with poor overall survival in patients with mesothelioma.	[93]
miR-126, miR-222, (Up)	Human (samples from malignant mesothelioma patients)	–	HUVECs, BEAS-2B, IMR-90, Met-5A	5 μg/cm^2^	EGFR, AKT, ERK, p38, PARP1	Exposed cells to asbestos via activating the EGFR pathway could increase the expression of mentioned miRs	[94]
miR-126 (Up), miR-520g (−), miR-222 (Up), miR-205 (−)	Human (non–small cell lung cancer patients)	–	–	–	–	Mentioned-miRNAs could be changed in lung malignancies caused by asbestos.	[20]
miR-197-3p (dysregulated)	Human	–	–	–		Serum levels of miR-197-3p could be dysregulated in workers exposed to asbestos.	[95]
miRNA profile, miR-197-3p, miR-1281, (Up)	Human (malignant pleural mesothelioma patients)	–	–	–	–	In workers who are ex-exposed to asbestos, the level of mentioned miRNAs increased.	[96]
miR-199/214 (Up)	specific pathogen-free F1 hybrid rats	–	MeT5A	–	Twist1, Akt, ERK, PTEN	In an animal model of sarcomatoid mesothelioma induced by asbestos, higher expression of miR-199/214 via targeting Twist1 could increase tumorigenesis.	[97]

### 2.4 Interaction between miRNAs and mercury

Exposure to mercury is regarded as a public health problem in the world. Hsa-miR-92a and hsa-miR-486 have been suggested as novel diagnostic markers for detection of occupational mercury poisoning. These two miRNAs have been found to be over-expressed in individuals exposed to occupational mercury. Over-expression of these miRNAs contributes to mercury toxicity through activation of NF-κB signaling via influencing expressions of KLF4 and Cezanne, respectively [98]. Another study has shown significant differences in the plasma levels of miR-124-3p, miR-125-5p, and miR-127-3p between patients with amalgam filling, dentists, and control group. Serum mercury concentration and plasma miR-125-5p and miR-127-3p levels have been positively correlated. Serum mercury has also been correlated with plasma miR-125-5p levels among dentists. This study shows the impact of amalgam filling in enhancement of serum mercury and plasma miRNA levels [99]. Besides, two distinct miRNA signatures have been reported to be activated upon neuronal differentiation and following MeHgCl-induced toxicity. Principally, exposure to MeHgCl could induce down-regulation of six out of the ten most up-regulated neuronal pathways in neural models. In fact, miRNAs expression profiling has been suggested as a possible way for evaluation of developmental neurotoxicity pathway [100]. Table 5 shows the interaction between miRNAs and mercury.

**Table 5:** Interaction between miRNAs and mercury.

miRNA	Human/animal Study	Dose	Cell line	Dose	Targets	Observations	Ref
hsa-miR-92a, hsa-miR-486, (Up)	Human (workers exposed to mercury)	–	293T, HUVECs	0–10 μM, 24 h	NF-κB, KLF4, COX-2	Both mentioned miRNAs via afecting NF-κB activity could lead to mercury toxicity.	[98]
miR-124-3p (−), miR-125-5p (Up), miR-127-3p (Up)	Human (amalgam filling patients and Dentists)	–	–	–	–	There is a relationship between serum mercury levels of miRNAs.	[99]
9 upregulated (such as miR-141, miR-196b), 5 downregulated (such as miR-217, miR-296)	–	–	H9, hESCs	0–500 nM	SOX2, FGF4, DNMT3B, COL2A1	Methyl Mercury could Induce neuronal toxicity and change the expression of miRNAs.	[100]

### 2.5 Interaction between miRNAs and chromium compounds

Hexavalent chromium [Cr(VI)] has been shown to induce various kind of cancer including lung cancer. Cr(VI) treatment can also increase expression of Nrf2, a redox sensitive transcription factor with protective effcets on normal cells. Mechanistically, expression of redox sensitive miRNAs miR-27a and miR-27b is ecreased after 1 month exposure to Cr(VI), leading to alteration sin levels of their target Nrf2. Taken together, suppression of miR-27a/b leads to up-regulation of Nrf2 at early and late stages of exposure to Cr(VI) [101]. Cr(VI) has also been found to induce malignant transformation in lung bronchial epithelium through ROS-dependent induction of miR-21-PDCD4 signals [102]. Table 6 shows the interaction between miRNAs and chromium compounds.

**Table 6:** Interaction between miRNAs and chromium compounds.

miRNA	Human/animal Study	Dose	Cell line	Dose	Targets	Observations	Ref
miR-21 (Up)	Human, female BALB/c mice, Athymic nude mice	1.2 mg/ml, once/week, 3 months	BEAS-2B, NL20, A549, H23, H2030, H460	0–5 μM, 24 h	PDCD4, β-catenin, c-Myc, TCF4, E-cadherin	Cr(VI) via upregulating miR-21 could lead to malignant transformation.	[102]
miR-27a/b (Down)	nude mice, BALB/cJ mice	zincchromate (1.0 mg/mL), 3 months	BEAS-2B	1 μM, 6 months	Nrf-2, KEAP1, HO-1	Overexpression of miR-27a/b via sponging Nrf-2 could act against Cr(VI) and lead to tumor suppression.	[101]
miR-223-3p, miR-327, miR-466f-3p, (Up)	C57BL mice offspring	0.14 and 1.19 mg Cr/kg	–	–	Akt1, Pik3ca	In adult mice offspring, maternal chromium restriction via targeting miRNAs could lead to insulin resistance.	[103]
9 downregulated miRNAs (such as miR-451, miR-301)	Human	–	–	–	–	Urinary chromium levels could change the expression of miRNAs in patients with cardiovascular diseases and metabolic diseases.	[104]
miR-494 (Down)	nude mouse	–	BEAS-2B	K_2_Cr_2_O_7_ (0.25 μM), 5 months	c-Myc	Cr(VI) via promoting c-Myc expression could lead to tumorigenesis.	[105]
miR-3940-5p (Down)	Human	–	–	–	XRCC2, BRCC3	In workers exposed to Cr(VI), miR-3940-5p could lead to genetic damage	[106]

### 2.6 Interactions between miRNAs and beryllium sulfate

The carcinogenic material beryllium sulfate (BeSO4) can affect expression of a number of non-coding RNAs in human bronchial epithelial cells. This substance has been found to up-regulate expression of 36 circRNAs and down-regulate other 35 circRNAs in these cells. Hsa_circ_0004214 and hsa_circ_0003586 have been among up-regulated circRNAs; and hsa_circ_0047958, hsa_circ_0001944, and hsa_circ_0008982 have been among down-regulated ones. These circRNAs can affect expression of a number of miRNAs that regulate cellular senescence, as well as TNF, NF-κB, HIF-1, and Hippo signaling pathways. The toxic effects of this substance is mainly mediated through sponging miR-663b and regulating JAK/STAT signaling [107]. Another study has shown that BeSO4 increases expression of some inflammatory molecules, including IL-10, TNF-α, IFN-γ, iNOS, and COX-2. Most notably, expression of 179 miRNAs has also been found to be changed by this substance. A number of these miRNAs have been shown to contribute to the transcription regulation, or modulation of MAPK, and VEGF signaling pathways [108].

### 2.7 Interaction between miRNAs and fluoride

Expression of several miRNAs has been shown to be changed in rat renal cortex following subchronic exposure to fluoride. These miRNAs have been mainly associated with extracellular matrix-receptor interactions, Mucin type O-glycan synthesis and Gap junctions. Moreover, expressions of miRNAs involved in cancer and proliferation have been changed after exposure to fluoride [109]. Combination of fluoride and aluminum (FA) has been shown to trigger apoptosis of rat hippocampal neurons and NG108-15 cells, enhance expression of miR-34b-5p, and decrease levels of Gnai2, PKA, ERK and CREB. Notably, suppression of miR-34b-5p expression could ameliorate FA-associated apoptosis and changes in the expressions of mentioned genes. Besides, miR-34b-5p has been found to modulate expression Gnai2 through targeting its 3′-UTR, indicating that miR-34b-5p participate in FA-associated neuron apoptosis through negatively targeting Gnai2 and suppressing activity of PKA/ERK/CREB cascade [110].

Furthermore, fluoride has been demonstrated to affect expression of 35 miRNAs, particularly those associated with glycolipid metabolism in the liver. In fact, these miRNAs could mediate fluoride-induced disturbance in the glycolipid metabolism, possibly through affecting activity of insulin, PPAR, and FOXO pathways [111].

### 2.8 Interaction between miRNAs and a combination of hazardous compounds

A number of studies have compared the effects of different hazardous materials in cell lines or animal models. For instance, experiments in C57BL/6J WT mice have shown that arsenic has more potent effects in disruption of the INS-1 beta cell miRNA landscape thansignature compared with cadmium or manganese [112]. Meanwhile, mixture of As–Cd–Pb has been shown to induce cellular transformation through affecting expression of miR-222 and post-transcriptional regulation of Rad51c levels [113]. Another study has assessed the association between miRNA profile in the cervix during pregnancy and levels of lead and mercury. This study has reported negative associations between levels of 17 miRNAs and toenail mercury levels. Moreover, tibial bone lead levels have been associated with down-regulation of miR-575 and miR-4286. Taken together, miRNAs levels in the human cervix has been suggested as novel markers for maternal exposures during pregnancy [114]. Table 7 shows the interaction between miRNAs and a combination of hazardous compounds.

**Table 7:** Interaction between miRNAs and a combination of hazardous compounds.

Compounds	miRNA	Human/animal Study	Dose	Cell line	Dose	Targets	Observations	Ref
Inorganic arsenic (iAs), manganese (Mn), CdCl2	miR-146a, (Up in iAS), (Down in CdCl2)	C57BL/6J WT mice	0.1 mg As/L	INS-1832/13,	iAs (1 μM), CdCl2 (5 μM), Mn (25 μM), 24 h	NF-κB, Camk2a	In INS-1832/13 cells (i.e. insulinoma cells), arsenic had more powerful damaging effects compared to others.	[112]
Pb, As, and CdCl2	miR-222 (Up)	–	–	Balb/c 3T3	NaAsO_2_ (2 μM), Pb (5 μM), and CdCl2 (2 μM)	Rad51c	A mixture of all hazardous materials could lead to cellular transformation via the miR-222/Rad51c axis.	[113]
Pb and Mercury	miR-575, miR-4286, (Down)	Human (pregnant women)	–	–	–	–	In the human cervix of pregnant women exposed to mercury and Pb, the miRNA expression was altered.	[114]
CdCl2 and NaAsO_2_	miR-let7a, miR-146a, (Down)	male Wistar rats	NaAsO_2_ (5 mg/kg), CdCl2 (1 mg/kg), one month	–	–	–	Arsenite and CdCl2 could lead to organ toxicity.	[115]

### 2.9 The effects of antioxidants on expression of miRNAs during exposure with hazardous compounds

**Fig. 3:**
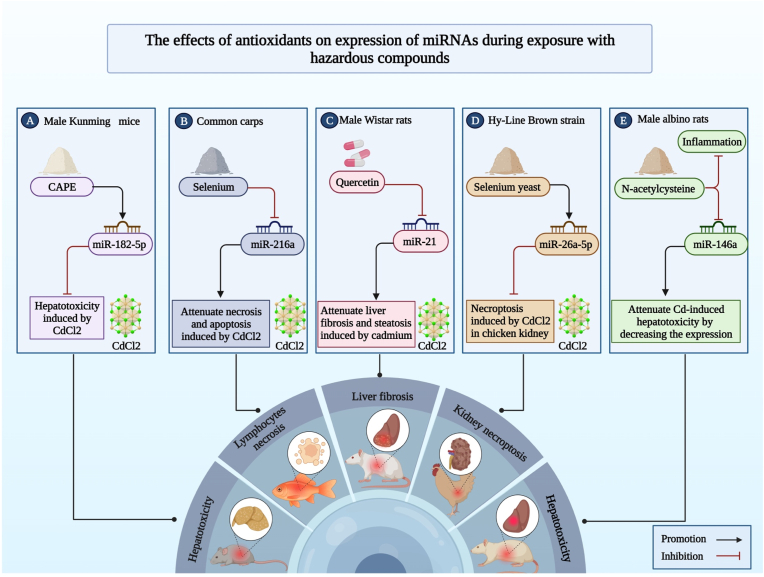
The illustration shows the effects of antioxidants on miRNA expression during exposure to hazardous compounds. (A) CAPE could downregulate hepatotoxicity induced by CdCl2 through upregulation of miR-182-5p. (B) Selenium in common carp lymphocytes may inhibit the promotion of miR-216a, necrosis, and apoptosis caused by CdCl2. (C) Quercetin, via inhibiting miR-21, could attenuate liver fibrosis and steatosis induced by cadmium. (D) Selenium yeast in the kidney of the chicken could protect against necroptosis caused by CdCl2 by increasing the expression of miR-26a-5p. (E) N-acetylcysteine in male albino rats could attenuate Cd-induced hepatotoxicity by decreasing the expression of miR-146a and inflammation.

Treatment with antioxidants can ameliorate the effects of hazardous materials on body organs through modulation of expression of miRNAs (Table 8). For instance, treatment with quercetin via inhibiting miR-21 could attenuate liver fibrosis and steatosis induced by cadmium [116]. Moreover, Se–Y via increasing the expression of miR-26a-5p could act against necroptosis induced by CdCl2 in the kidney of the chicken [117] (Fig. 3). Other examples are shown in Table 8.

**Table 8:** Interaction between miRNAs, hazardous compounds, as well as antioxidants.

Type	miRNA	Human/Animal Study	Dose	Other treatments (Source of antioxidants)	Cell line	Dose of hazardous materials	Targets or Pathways	Results	Ref
CdCl2	miR-21a (Up)	Male Wistar rats	10 moml/L, 5 months	Quercetin; 50 mg/kg, 5 months	–	–	NF-kB P65, Nrf2, Smad3, SREBP1, TGF-β1	Treatment with quercetin via inhibiting miR-21 could attenuate liver fibrosis and steatosis induced by cadmium.	[116]
CdCl2	miR-26a-5p (Down)	Hy-Line Brown strain	150 mg/kg, 42 days	Selenium yeast (Se–Y); 0.5 mg/kg, 42 days	–	–	HSP60/80/90, PTEN, PI3K/AKT, RIP1/3	Se–Y via increasing the expression of miR-26a-5p could act against necroptosis induced by CdCl2 in the kidney of the chicken.	[117]
CdCl2	miR-30a (Down)	Hy-Line Brown chickens	150 mg/kg, 3 months	Se; (0.2 mg/kg of Na2SeO3), 3 months	–	–	GRP78, JNK, IRE-1, ATG5, LC-3I/II, Beclin-1	In the chicken kidneys, CdCl2 via mediating GRP78 and miR-30a could cause JNK-dependent autophagy.	[118]
CdCl2	miR-125a, miR-125b, (Down)	–	–	Selenium; 5–20 μM, 0.5 h before the Cd administration	LLC-PK1	20 μM, 12 h,	Bax, Bak, Caspase-3	Treatment with selenium via targeting miR-125a/b could inhibit apoptosis induced by CdCl2.	[119]
CdCl2	miR-146a (Up)	Male albino rats	3 mg/kg, daily, 2 months	N-acetylcysteine (NAC); 100 mg/kg, daily, 2 months	–	–	NF-κB p65, TNF-α, IL-1β, TRAF6	NAC could attenuate Cd-induced hepatotoxicity by decreasing the expression of miR-146a and inflammation.	[120]
CdCl2	miR-182-5p (Down)	male Kunming mice	1.5 mg/kg,	CAPE; 10 μmol/kg body weight	–	–	TLR4, IL-1β, IL-6, TNF-α, PI3K/AKT, mTOR, Caspase-3	CAPE could downregulate hepatotoxicity induced by CdCl2.	[121]
CdCl2	miR-216a (Up)	common carps	–	Se; (10^−6^ mol/L of Na2SeO3), 6 h	Lymphocyte	4 × 10^−5^ mol/L, 6 h	PI3K/AKT, Bax, Bcl-2, Caspase-3/9, RIP3, MLKL	Se could act against the promotion of the miR-216a, necrosis, and apoptosis induced by CdCl2 in the lymphocytes of common carp.	[122]
CdCl2	miR-661 (Down)	–	–	Caffeic acid phenethyl ester (CAPE); 10 μM	HepG2	0–30 μM, 24 h	Caspase-9	CAPE could downregulate apoptosis induced by CdCl2.	[123]
Pb	miR-16-5p (Up)	Hy-Line Brown chickens	–	Se; (1 μM of Na2SeO3)	Neutrophil	12.5 μM	IGF1R, PiK3R1, p53, Bcl-2, Bax, Caspase-3/8/9	In chicken neutrophil cells, Se via targeting miR-16-5p had an antagonistic impact against lead-induced apoptosis.	[73]
Pb	miR-224 (Up)	Male Wistar rats	30 mg/kg, once every 2 days, less than 4 months	Selenium nanoparticles (Se-NPs); 0.5 mg/kg, less than 4 months	–	–	ID1	Se-NPs via inhibiting miR-224 could attenuate adverse effects of Pb on thyroid tissues.	[124]
ATO	miR-182-5p (down)	–	–	NAC; 10 mM, 4h	U87MG, S1 GBM primary cells, A549, H1299	0–5 μM	SESN2, HO-1	ATO via inhibiting miR-182-5p and increasing SESN2 could impede oxidative stress.	[125]

## 3 Conclusions

Several compounds have been shown to affect expressions of miRNAs, thus disturbing activity of several signaling pathways in different tissues and contributing to diverse disorders. The impacts of the environmental exposure to hazardous materials on the epigenome have attracted a substantial interest in the recent years. miRNAs as important regulators of gene expression are of considerable importance in this regard. Several miRNAs have been shown to be dysregulated during exposure to these toxic agents being responsible for alterations in the physiological processes after exposure to toxins. Therefore, expression profiling of miRNAs represents a possible route for determination of the effects of hazardous materials on the body organs. Since miRNAs are stable in the circulation and are protected from endogenous RNase, miRNAs are regarded as suitable blood-based biomarkers not only for detection of human diseases but also for estimation of the amount of exposure to hazardous materials. However, the underlying mechanisms of contribution of miRNAs in toxic effects of these materials have not been elucidated yet.

More research is needed to establish a reliable profile of miRNA alterations after exposure to each hazardous material. These putative well-defined miRNA signatures can be used for early detection of disorders being associated with these compounds. Examples of these disorders include cancers, neurodegenerative disorders and pulmonary disorders. In addition, identification of the altered miRNAs during exposure to toxins can help in design of novel therapeutic modalities for complex disorders that are associated with environmental exposure. Finally, certain antioxidants have been found to ameliorate the effects of hazardous materials, particularly CdCl2 and Pb on miRNAs profile, thus amending the organ impairment/dysfunction associated with hazardous materials. Future high throughput studies are needed to find the suitable antioxidant for amelioration of each condition. These antioxidants are expected to reverse the effects of these materials on body organs; thus, they can be prescribed for persons that environmental or occupational exposure to hazardous materials. The off-target effects of antioxidants should be assessed in future studies.

## Ethics approval and consent to participant

Not applicable.

## Consent of publication

Not applicable.

## Funding

Not applicable.

## Authors’ contributions

SGF wrote the draft and revised it. MT and EJ designed and supervised the study. SRA, BMH, SDO and HS collected the data and designed the figures and tables. All the authors read the submitted version and approved it.

## Declaration of competing interest

The authors declare they have no conflict of interest.

## Abbreviations

TEAD1: TEA domain transcription factor 1
PIM1: Pim-1 proto-oncogene
NQO1: NAD(P)H quinone oxidoreductase 1
hERG: human ether-a-go-go-related gene
SATB2: special AT-rich sequence-binding protein 2
SP1: Specificity Protein 1
PHLPP: PH domain leucine-rich repeat protein phosphatase
LEF1: lymphoid enhancer factor
TCF1: T-cell factor 1
XIAP: X-linked inhibitor of apoptosis protein
CCL20: CC chemokine ligand 20
CCM3: cerebral cavernous malformation 3
PLD1): Phospholipase D1
Camk2a: Ca2+/calmodulin-dependent protein kinase IIα
KLF4: Krüppel-like factor 4
COX-2: cyclooxygenase-2
PDCD4: Programmed cell death 4
IGF1R: insulin-like growth factor 1 receptor
ID1: Iodothyronine deiodinase type 1
SESN2: Sestrin-2
HBEC: human bronchial epithelial cells
hBMSCs: human bone marrow mesenchymal stem cells
BMECs: bovine mammary epithelial cells
HUVECs: Human Umbilical Vein Endothelial Cells
ATO: Arsenic trioxide
NaAsO₂: Sodium arsenite
AsCl3: Arsenic chloride
As4S4: Arsenic sulfide
CdCl2: Cadmium
Pb: Lead
Cr: chromium
Cr(VI): Hexavalent chromium
